# Antidiabetic, Antihyperlipidemic and Antioxidant Activities of a Novel Proteoglycan from *Ganoderma Lucidum* Fruiting Bodies on db/db Mice and the Possible Mechanism

**DOI:** 10.1371/journal.pone.0068332

**Published:** 2013-07-11

**Authors:** Deng Pan, Dang Zhang, Jiasheng Wu, Congheng Chen, Zhixue Xu, Hongjie Yang, Ping Zhou

**Affiliations:** 1 State Key Laboratory of Molecular Engineering of Polymers, Department of Macromolecular Science, Fudan University, Shanghai, P. R. China; 2 Yueyang Hospital of Integrated Traditional Chinese and Western Medicine, Shanghai University of Traditional Chinese Medicine, Shanghai, P. R. China; 3 Pharmacy College, Shanghai University of Traditional Chinese Medicine, Shanghai, P. R. China; Consiglio Nazionale delle Ricerche, Italy

## Abstract

Previously, we screened a proteoglycan for anti-hyperglycemic, named *FYGL*, from *Ganoderma Lucidum*. For further research of the antidiabetic mechanisms of *FYGL in vivo*, the glucose homeostasis, activities of insulin-sensitive enzymes, glucose transporter expression and pancreatic function were analyzed using db/db mice as diabetic models in the present work. *FYGL* not only lead to a reduction in glycated hemoglobin level, but also an increase in insulin and C-peptide level, whereas a decrease in glucagons level and showed a potential for the remediation of pancreatic islets. *FYGL* also increased the glucokinase activities, and simultaneously lowered the phosphoenol pyruvate carboxykinase activities, accompanied by a reduction in the expression of hepatic glucose transporter protein 2, while the expression of adipose and skeletal glucose transporter protein 4 was increased. Moreover, the antioxidant enzyme activities were also increased by *FYGL* treatment. Thus, *FYGL* was an effective antidiabetic agent by enhancing insulin secretion and decreasing hepatic glucose output along with increase of adipose and skeletal muscle glucose disposal in the late stage of diabetes. Furthermore, *FYGL* is beneficial against oxidative stress, thereby being helpful in preventing the diabetic complications.

## Introduction

Chronic metabolic disorder is considered to be the major leading causes of death in the world. In particular, diabetes mellitus (DM), the most common form of metabolic dysfunction and characterized by absolutely or relatively insufficient in insulin secretion or insulin action [1], currently affects more than 346 million people worldwide, with almost 3.4 million mortalities. The World Health Organization (WHO) estimated that DM is responsible for approximately 5% of all deaths worldwide and predicted more than 50% increase in the diabetes-related mortality in 10 years [2]. Nowadays the agents used for diabetes treatment mainly are synthetic drugs and insulin. However, these drugs usually come with considerable side effects, such as hypoglycemia, drug-resistance, dropsy and weight gain [3]. Apart from those currently available therapeutic options for diabetes, herbal medicines have been recommended by WHO for the treatment of diabetes [4]. Herbal remedies are relatively effective, cheap and almost no side effects, compared to synthetic agents [5]. One of examples is *Ganoderma lucidum,* a white rot fungus, which has been widely used as a tonic in promoting longevity and health for thusands years [6]. Recently, a great deal of work has been carried out on this fungus, indicating that some ingredients from *G. lucidum* can promote the release of serum insulin and decrease the plasma glucose concentration *in vivo* [7]–[10].

In the insulin signaling pathway, there is one important phosphatase, protein tyrosine phosphatase 1B (PTP1B) considered to play an important role in the intracellular signal transduction process and metabolism [11]. Previously, we successfully isolated a highly efficient PTP1B inhibitor, named *FYGL* (Fudan-Yueyang-*G. lucidum*), from the fruiting bodies of *G. lucidum* [12]. *FYGL* was capable of decreasing the plasma glucose level and enhancing the insulin sensitivity *in vivo*. We have already investigated the dominant components, inhibition kinetics, pharmacology and toxicity of *FYGL in vivo*, and demonstrated that *FYGL* could serve as a drug candidate or a health-care food for the diabetic therapy or protection with high safety [12]–[14].

However, many phytochemicals were reported to have multiple functions *in vivo* [15], [16] and due to the complex pathogenesis of DM, in present work we focus on the relationship between oxidative stresses, glucose transport and DM which has been appreciated for a long time, although the exact mechanism on how those factors cause DM is still unclear [17]. Thus, for research of the possible antidiabetic mechanisms of *FYGL in vivo*, the glucose homeostasis, activities of insulin-sensitive enzymes, relevant glucose transporter expression and pancreatic function were analyzed with db/db mice, a kind of obesity rodent mode for DM. Furthermore, *FYGL* protection from the oxidative damage induced by diabetes and its role in ameliorating the development of diabetes were also investigated.

## Materials and Methods

### Ethics Statement

All animal trial procedures instituted and approved by Ethical Committee for the Experimental Use of Animals in Center for Drug Safety Evaluation, Shanghai University of Traditional Chinese Medicine were followed. All animals were housed in plastic cages (4 mice/cage) with free access to drinking water and a pellet diet, under controlled conditions of humidity (50±10%), light (12/12h light/dark cycle) and temperature (23±2°C). At the end of the treatment, mice were fasted over night, anaesthetized and killed by cervical decapitation.

### Preparations of *FYGL*

The preparation procedures were based on a previous work [12]. Briefly, after the dried fruiting bodies of *G. lucidum* was milled and defatted with boiling ethanol, the residues was decocted with boiling water. The supernatant of the decocted mixture was discarded, and the solid residues were extracted by ammonia aqueous solution at room temperature. The supernatant extracted from the aqueous solution was neutralized, concentrated, dialyzed and lyophilized successively; the crude extract was then collected. Subsequently, the crude extract was dissolved in distilled water and further purified by Sephadex G-75 column (l.d. 80×26 cm) chromatography with Sodium chloride solution as the eluent. The eluted fractions were monitored by the phenol-sulfuric acid method with ultraviolet (UV) absorption at 490 nm and the main fraction, named as *FYGL,* was collected.

### Animal Trials

C57BL/6 mice and C57BL/6 db/db mice (male,6 weeks old) were obtained from Shanghai Institute of Material Medical, Chinese Academy of Sciences and housed individually in plastic cage at 25°C. Ten C57BL/6 mice were set as vehicle (Group I, normal mice). The DM C57BL/6 db/db mice were confirmed by the symptoms of hyperglycemia, polyphagia, polydipsia, and polyuria. After acclimatized for 2 weeks, only those animals with plasma glucose higher than 11.1 mmol/L were selected as diabetic model for the following experiments. A total of sixty animals at the age of 8 weeks old, including 10 normal mice and 50 db/db DM mice, were selected and divided into six groups (numbered as groups I–VI) with 10 mice in each group. Group I were normal mice treated with 0.9% saline solution (normal); Group II to VI were DM mice treated with 0.9% saline solution (control), 75 mg/kg *FYGL* (low dosage) 250 mg/kg *FYGL* (middle dosage), 450 mg/kg *FYGL* (high dosage), and 200 mg/kg metformin (positive), respectively. All drugs were dissolved in 0.9% saline and administered orally for 8 weeks. The effects of *FYGL* in mice were determined by measuring weekly the body weigh and fast blood glucose concentration (FBG) which was collected via the tail vein. The dosage of *FYGL* and metformin was adjusted weekly according to the body weight to maintain the similar dose per kilogram of mice over the entire experiment.

The oral glucose tolerance test (OGTT) was carried out after 8 weeks treatment [18]. After animals were fasted for 16 h, they were fed glucose solution (1.0 g/kg) orally, and 2 h late, the blood samples were collected via tail vein for the measurement of postprandial 2 h blood glucose levels.

After 8 weeks treatment, i.e., at the age of 16 weeks old, all animals were sacrificed and 2 ml blood samples were collected from eye artery, and then centrifuged at 4000 r/min for 10 minutes to separate serum which was then frozen at −70°C. The liver, skeletal muscle and adipose tissue were quickly removed and frozen at −70°C. The isolated pancreas tissues were fixed in 10% (v/v) neutral buffered formalin.

### Biochemical Parameters Measurements

The FBG concentration of whole blood obtained from the tail veins was measured weekly by a glucose analyzer (Sigma Diagnostics, St. Louis, MO). The glycosylated hemoglobin (HbAlc) level at 1 and 8 weeks treatments was measured with analyzer (Roche Diagnostics, Basel, Switzerland) using whole blood obtained from eye artery. The serum insulin, C-peptide, glucagons, and leptin levels were measured by radio-immunoassay (RIA) method. The serum and hepatic glucokinase (GK) and phosphoenolpyruvate carboxykinase (PEPCK) were measured by the ELSIA reagent kits purchased from Nanjing Jianchen Bioengineering Institute (Nanjing, China).

### Lipids and Hepatic Glycogen Assay

The lipids of hepatic tissue were extracted by chloroform/methanol mixture (2∶1 v/v) on the method of Folch et al. [19]. Total lipids contents in liver were quantified gravimetrically using a lyophilizer (Sihuang, LGJ-10C, Shanghai, China). The dried hepatic lipid residues were dissolved in 1 ml absolute ethanol for cholesterol and triacylglycerol assays. Lipid profiles, including total cholesterol (TC), triacylglycerol (TG), low-density lipoprotein-cholesterol (LDL-C) and high-density lipoprotein-cholesterol (HDL-C), in liver and serum were measured by the commercial enzymatic kits purchased from Nanjing Jianchen Bioengineering Institute (Nanjing, China). The atherogenic index (AI) was calculated according to the Friedewald equations as following [20]:
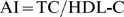



The hepatic glycogen content was determined by using an anthrone reagent (2 g anthrone and 1 L 95% (v/v) H_2_SO_4_) and measured by UV at 620 nm [21]. In brief, the liver tissues were homogenized and dissolved in 2 M NaOH solution at 100°C for 30 min.

### Hydrogen Peroxide and Hepatic Peroxidation Assay

The hydrogen peroxide levels in liver were measured by Wolff’s method [22]. The hepatic malondialdehyde (MDA) concentrations were measured by UV spectrometer on the method of Ohkawa [23]. The hepatic protein carbonyl (PC) contents were estimated on the basis of formation of protein hydrazone which can be measured at 366 nm on the method of Levine [24]. Hepatic 8-hydroxydeoxyguanosin (8-OHdG) level was estimated by EILSA method, using commercial kits from Nanjing Jianchen Bioengineering Institute (Nanjing, China).

### Antioxidant Enzyme Activities in Serum and Liver

Super oxide dismutase (SOD) activity was measured at UV of 420 nm using the inhibition of pyrogallol autoxidation for 10 min according to the method of Marklund [25]. The activity of enzyme was determined by that which inhibited the oxidation of pyrogallol by 50%. Catalase (CAT) activity was determined according to Abei’s method by decomposing H_2_O_2_ for 5 min and monitored at 240 nm [26]. Glutathione peroxidase (GSH-px) was assayed by the method of Paglia [27].

### Western Blot Analysis

The livers, skeletal muscles and adipose tissues were prepared for western blot analysis according to the literature report [28]. In brief, the tissues were homogenized with a buffer containing 150 mmol/L NaCl, 10 mmol/L sodium pyrophosphate, 10 mmol/L NaF, 2 mmol/L acetic acid, 2 mmol/L phenylmethylsulfonyl fluoride (PMSF), 5 mg/L leupeptin, 1% Nonidet P-40, and 10% glycerol. The homogenates were centrifuged (12000×g, 15 min) at 4°C. The protein concentrations in the supernatant homogenate were measured with Bradford protein assay reagent, using bovine serum albumin (BSA) as standard. The supernatant homogenates containing 20 µg protein were run on SDS-PAGE (10% gel) and transferred electrophoretically onto the nitrocellulose (NC) membrane. The supernatant homogenates from liver were used for GLUT2 assay, while the supernatant homogenates from skeletal muscle and adipose were used for GLUT4 assay. The NC membranes were then blocked for 2 h at room temperature and then incubated with rabbit anti-mouse GLUT2 (1∶50000; Biogenesis, Sandown, NJ) and anti-mouse GLUT4 (1∶50000; Biogenesis, Sandown, NJ). The NC membrane were then washed for 30 min with wash solution, followed by 1 h incubation with anti-rabbit IgG conjugated with the horseradish-peroxidase in block solution. Finally, the NC membranes were washed for 30 min with wash solution, and the immunoreactive bands were detected by the enhanced chemiluminescence’s method.

### Pancreas Histological Observation

The pancreas tissues were fixed in 10% (v/v) neutral buffered formalin, processed routinely, and embedded in paraffin wax. Paraffin sections were cut into 5 µm thickness and deparaffinized in xylene for 5 min dehydrated through the graded ethanol. The sections were stained with hematoxylin and eosin (H&E) for histopathological analysis by photomicroscope. The total operative procedures was complied with the standard protocols and the examination of slides was performed by a pathologist in a blind to the experimental profile.

### Statistical Analysis

The results were represented as mean ± SD. Data were analyzed by a statistical analysis program. Comparison between control and treated groups was analyzed by Student’s t-test and the significant difference was established by ANOVA variance analysis. Differences of *p*<0.05 were considered statistically significant.

## Results

### The Basic Structural Properties of *FYGL*

Component analysis indicated that *FYGL* was composed of proteoglycan with a viscosity-average molecular weight of 2.6×10^5^ [12]. Based on a preliminary work, the polysaccharide moiety in *FYGL* mainly consisted of 1,2-linked α-L-rhamose, 1,2,4-linked α-L-rhamose, 1-linked α-D-glucose, 1,2,3,4-linked α-D-glucose, 1,4-linked α-D-glucose, 1,2,4-linked α-D-glucose, 1,6-linked β-D-glucose, 1,6-linked β-D-galactose, 1,3,6-linked β-D-galactose and 1-linked β-D-galactose. The analysis of amino acids in *FYGL* indicated that there were 16 common amino acids, among which aspartic acid, glycine, serine, alanine, glutamic acid and threonine were the dominant components. And more research demonstrated that the protein moiety in *FYGL* covalently linked to the 1,3-linked α-D-glucose and 1,6-linked β-D-glucose residue of polysaccharide in O-linkage type via both serine and threonine (Data unpublished and detailed structure of *FYGL* in supporting information for review only).

### Effects of *FYGL* on Body Weight, FBG, OGT and HbAlc

**Figure 1:**
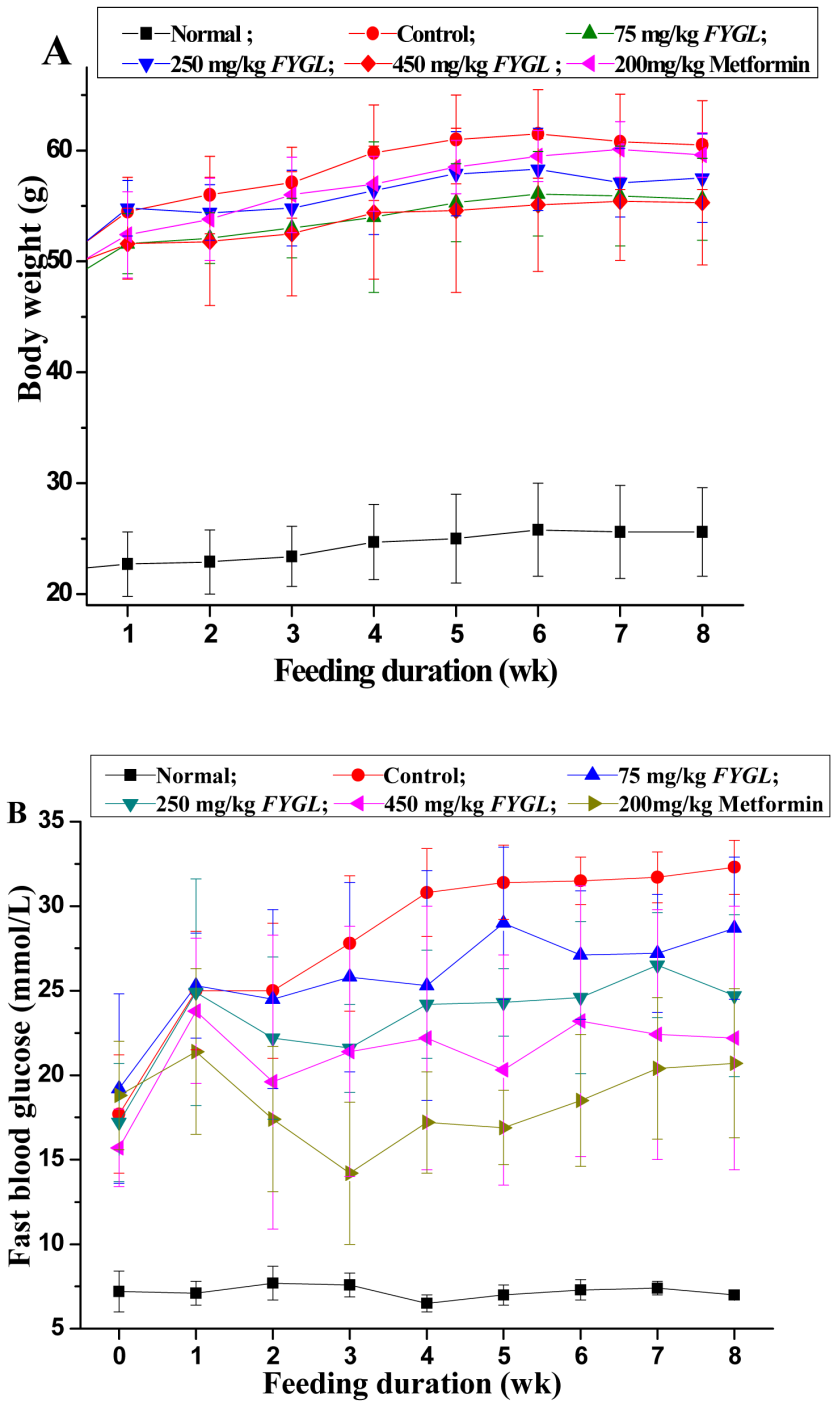
Body weight and blood glucose. Effect of *FYGL* and metformin on normal and db/db diabetic mice for 8 weeks, Results are expressed as mean ± S.E.M., n = 10, (A) Body weight changes (B) Fast blood glucose (FBG).

Changes in the body weight of all the studied mice during 8 weeks period were shown in Figure 1A. The body weight of all groups of diabetic mice, including the *FYGL*-treated, metformin-treated and control goups, increased throughout the experimental period, whereas that of the normal groups of mice was quite stable without too much alteration. However, when compared with controls, the body weight of mice treated with *FYGL* and metformin for 8 weeks seems dose-dependently decreased (without significant difference).

As shown in Figure 1B, all db/db mice were diabetic when the experiment began, as indicated by the FBG concentration and the dose-dependently decreases of FBG concentration was observed after 4 weeks of treatment with *FYGL* and metformin compared with the control group.

**Figure 2:**
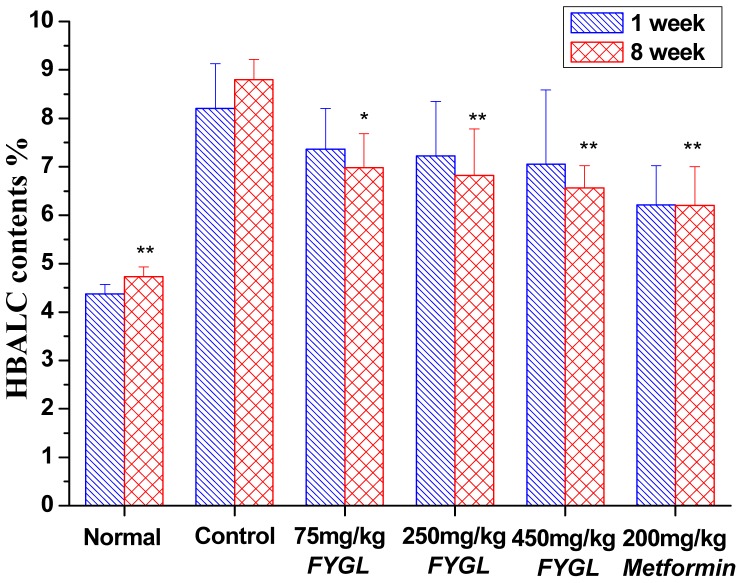
The glycated hemoglobin levels. The glycated hemoglobin (HbAlc) levels of normal and db/db diabetic mice for 1 and 8 weeks. Results are expressed as mean ± S.E.M., n = 10, *p<0.05 as compared to diabetic control and **p<0.01 as compared to diabetic control.

As shown in Figure 2, after 8 weeks treatments, the HbA1c level significantly decreased from 8.8±0.42% to 6.98±0.16% (*p*<0.05), 6.82±0.96% (p<0.01), 6.56±1.46% (p<0.05), and 6.2±0.8% (p<0.05), respectively, for the mice treated with low, middle, high-dosage *FYGL* and metformin, compared with that of diabetic control group.

**Figure 3:**
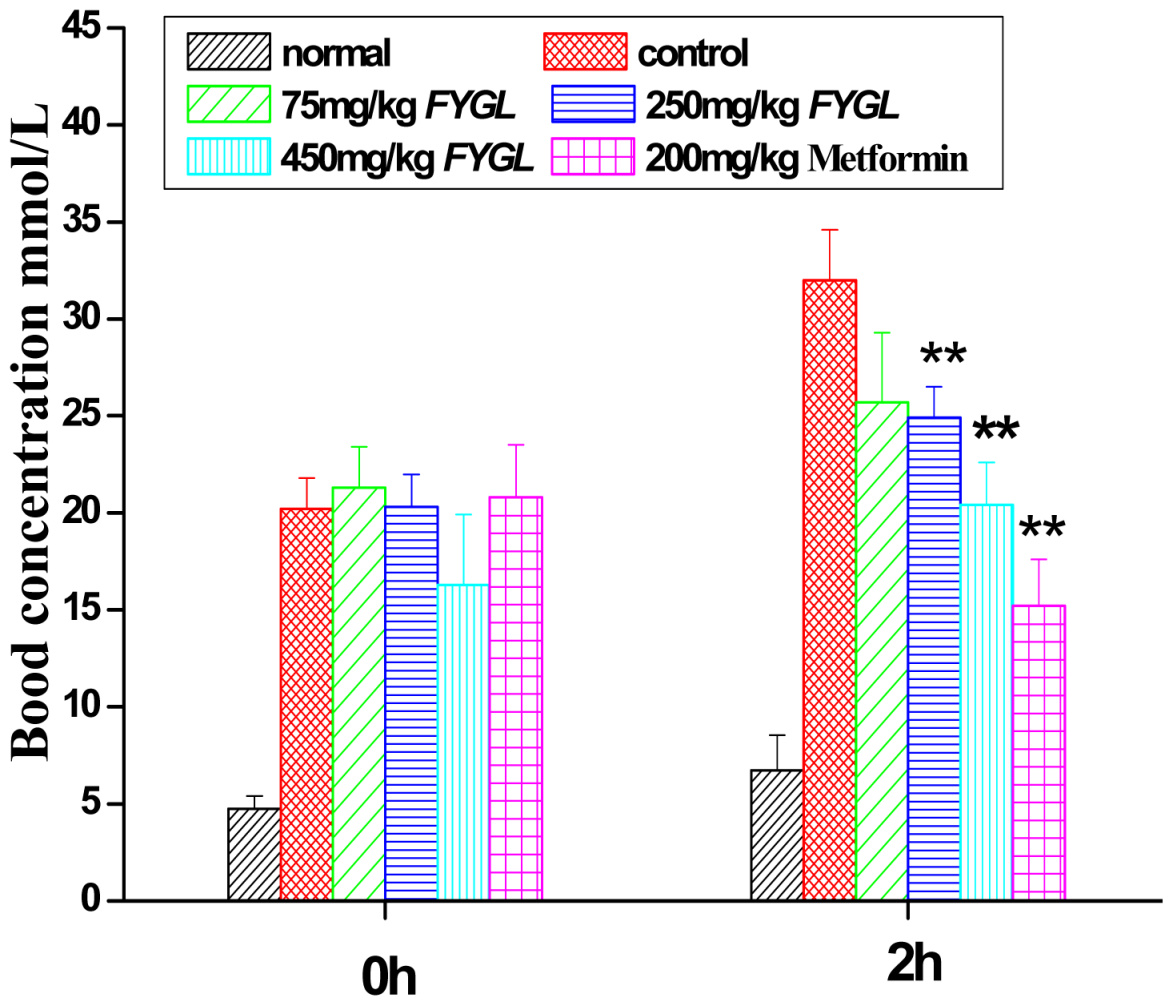
The postprandial 2 h glucose level. The postprandial 2 h glucose level normal and db/db mice after treatment for 8 weeks. Results are expressed as mean ± S.E.M., n = 10, *p<0.05 as compared to diabetic control and **p<0.01 as compared to diabetic control.

As shown in Figure 3, the postprandial 2 h blood glucose levels were 25.6±10.3 mmol/L and 21.4±9.3 mmol/L for groups with high-dosage *FYGL* and metformin treatments, respectively, which were significantly (*p*<0.01) lower than 34.0±2.6 mmol/l for control group after 8 weeks treatment. Hence, it was suggested that *FYGL* and metformin treatment could improve the oral glucose tolerance (OGT) for db/db mice.

### Effects of *FYGL* on Serum Insulin, C-peptide, Glucagon and Leptin Levels

As summarized in Table 1, the serum insulin level was increased in dose-dependent manner from 3.26±0.24 mU/L for the diabetic control group to 3.59±0.15, 3.69±0.13, 3.74±0.17 and 3.43±0.11 mU/L for the mice treated with low, middle, high-dosage *FYGL* and metformin, respectively. Similarly,the serum C-peptide level was increased from 11.88±0.71 ng/ml for diabetic control group to 13.55±0.65, 14.93±0.63, 15.22±0.83 and 15.19±1.18 ng/ml, respectively, for drug treated mice. Meanwhile, the leptin level was increased from 1.79±0.13 ug/L for diabetic control group to 1.93±0.97, 1.96±0.97, 2.04±0.24 and 1.97±0.09 ug/L, respectively, for drug treated mice. However, the serum glucagon level was decreased from 15.75±0.67 ng/L for control mice to 15.43±0.54, 15.04±0.19, 14.75±0.46 and 15.19±0.21 ng/ml, respectively, for drug treated mice.

**Table 1:** Levels of serum insulin, C-peptide, glucagon, leptin, blood glycosylated hemoglobin, and hepatic glycogen in normal and db/db mice after treatment for 8 weeks.

	Normal	Control	75 mg/kg *FGYL*	250 mg/kg *FYGL*	450 mg/kg *FYGL*	200 mg/kg Metformin
Insulin (mU/L)	3.67±0.16**	3.26±0.24	3.59±0.15*	3.69±0.13**	3.74±0.17**	3.43±0.11*
C-peptide (ng/mL)	15.69±0.64**	11.88±0.71	13.55±0.65	14.93±0.63**	15.22±0.83**	15.19±1.18**
Leptin (ug/L)	1.99±0.14*	1.79±0.13	1.93±0.97	1.96±0.97*	2.04±0.24*	1.97±0.09*
Glycosylated hemoglobin (%)	4.73±0.2**	8.80±0.42	6.98±0.7*	6.82±0.96**	6.56±.1.46*	6.20±0.8*
Glucagon (ng/L)	14.75±0.15**	15.75±0.67	15.43±0.54	15.04±0.19*	14.75±0.46**	15.19±0.21*
Glycogen (mg/g liver)	134±5.71**	54.5±6.82	64.8±13.70*	73.9±1.8**	76.3±2.4**	60±2.3**

Results are expressed as mean ± S.E.M., n = 10.

* p<0.05 as compared to diabetic control and **p<0.01 as compared to diabetic control.

### Effects of *FYGL* on Lipids Profile and Hepatic Glycogen

The effects of *FYGL* on serum and hepatic lipid profile were summarized in Table 2. TG and TC were significantly decreased by 47.8% (p<0.01) and 41.0% (p<0.01) in serum, and 33.6% (p<0.01) and 55.5% (p<0.05) in liver for high dose of *FYGL*-treated db/db diabetic mice, compared with those for the control. Furthermore, the serum LDL-C levels for both middle and high-dosage of *FYGL*-treated mice were significantly (p<0.01) lower than those for control group, whereas the serum HDL-C level for those drug-treated groups were higher (p<0.01) than that for control group. The atherogenic index (Al) value and LDL-C/HDL-C ratio were also significantly decreased for *FYGL*-treated group in dose-dependent manner, compared to that for control group.

**Table 2:** The activities of serum and hepatic antioxidant enzyme and the levels of hydrogen peroxide (H_2_O_2_), malondialdehyde (MDA), protein carbonyl (PC) and 8-hydroxydeoxyguanosin (8-OHdG) contents and in normal and db/db mice after treatment for 8 weeks.

	Normal	Control	75 mg/kg *FGYL*	250 mg/kg *FYGL*	450 mg/kg *FYGL*	200 mg/kg Metformin
**Serum**						
SOD (U/ml)	166.25±5.88**	148.2±4.46	150.87±7.6	157.98±8.65*	170.74±8.74*	161.67±6.84*
CAT (U/ml)	27.03±0.86**	25.79±1.26	25.91±0.93	26.76±0.61*	27.66±0.96*	26.76±0.89*
GSH-px (U/ml)	15.27±2.69**	13.42±2.01	15.20±3.65*	16.20±1.25*	17.06±1.99**	17.99±2.75**
**Liver**						
SOD (U/mg protein)	9.64±0.96**	6.04±0.18	6.24±0.19*	7.56±0.34**	8.18±0.23**	6.53±0.18*
CAT (U/mg protein)	10.21±1.50**	4.66±1.87	9.70±1.17*	10.63±1.07**	11.05±1.06**	10.02±1.76**
GSH-px (U/mg protein)	52.07±2.07**	35.17±6.57	37.86±3.7	50.94±6.25*	52.23±6.38*	47.50±1.62*
H_2_O_2_ (nmol/mg protein)	20.76±1.35**	36.01±3.88	30.21±2.10	27.06±1.65*	21.18±2.52*	28.49±3.25*
MDA(nmol/mg protein)	0.407±0.09**	1.40±0.07	1.11±0.06**	0.967±0.08**	0.507±0.08**	0.509±0.06**
PC (nmol/mg protein)	190.97±3.30**	334.62±13.23	260.53±12.3**	228.65±8.79**	213.69±11.03**	289.70±15.03*
8-OHdG (pg/mg protein)	897.73±25.12**	1828.89±17.70	1344.30±27.99*	1326.10±26.74*	1090.18±33.91**	1444.2±26.17*

Results are expressed as mean ± S.E.M., n = 10.

* p<0.05 as compared to diabetic control and **p<0.01 as compared to diabetic control.

### Effects of *FYGL* on Antioxidation Status

Hydrogen peroxide (H_2_O_2_), the main source of reactive oxygen species (ROS), was measured to preliminarily estimate the degree of oxidative stress. As shown in Table 3, high-dose *FYGL* and metformin treatments significantly (p<0.01) decreased the concentration of H_2_O_2_ in liver by 41.2% and 20.1%, respectively. Meanwhile, Lipid, protein and DNA damage in terms of malondialdehyde (MDA), protein carbonyl (PC) and 8-hydroxydeoxyguanosin (8-OHdG) contents were increased significantly (p<0.01) in diabetic control mice, as compared to those in normal control animals. However, middle and high-dosage *FYGL* treatments significantly (p<0.01) lowered the MDA, PC and 8-OHdG content in liver, as compared to the diabetic control group.

**Table 3:** Serum and hepatic lipid profiles in normal and db/db mice treated with *FYGL* for 8 weeks.

	Normal	Control	75 mg/kg *FGYL*	250 mg/kg *FYGL*	450 mg/kg *FYGL*	200 mg/kg Metformin
**Serum**						
TG (mg/ml)	0.77±0.09**	1.59±0.18	1.25±0.21**	1.08±0.25**	0.83±0.12**	0.94±0.06**
TC (mg/ml)	0.69±0.14**	1.34±0.15	1.05±0.08*	0.98±0.06*	0.79±0.08*	0.88±0.23*
LDL-C (mg/ml)	0.14±0.03**	0.85±0.11	0.62±0.08	0.43±0.06**	0.26±0.04**	0.36±0.02**
HDL-C (mg/ml)	0.40±0.05**	0.21±0.02	0.26±0.06	0.34±0.07*	0.38±0.04*	0.34±0.05*
AI	1.73±0.19**	6.38±1.18	4.04±0.71*	2.88±0.65**	2.08±0.22**	2.26±0.26**
LDL-C/HDL-C ratio	0.35±0.04**	4.04±1.23	2.38±0.12**	1.26±0.06**	0.68±0.09**	1.06±0.06**
**Liver**						
TG (mg/g)	7.86±1.14**	12.97±1.51	10.07±1.26*	8.87±1.94*	8.61±2.61*	10.54±1.06*
TC (mg/g)	8.12±2.25**	13.61±0.82	9.36±3.77*	6.75±3.40**	6.05±1.91**	6.27±1.37**

Results are expressed as mean ± S.E.M., n = 10 *p<0.05 as compared to diabetic control and **p<0.01 as compared to diabetic control.

AI = TC/HDL-C.

Table 3 depicts the activities of enzymatic antioxidants in both liver and serum of all groups. In diabetic group, the activities of SOD, CAT and GSH-px in liver were significantly decreased as compared to that in the normal group. Administration of *FYGL* improved the activities of these enzymes in diabetic mice in a dose-dependent manner. For instance, the high-dosage *FYGL* treatment significantly (p<0.01) increased the activity of SOD, CAT and GSH-px in liver by 35.4%, 137% and 48.5% as compared to that of control mice.

### Effects of *FYGL* on Activities of Glucose-regulating Enzyme

As summarized in Table 4, the hepatic PEPCK activities in diabetic control mice were increased by 144.9% (p<0.01), while the hepatic GK activities were decreased by 43.3% (p<0.01), compared to that in normal mice. However, middle and high-dosase *FYGL* treatment significantly elevated GK activities by approximately 25.5% (p<0.05) and 64.5% (p<0.01) compared with that of the diabetic control group. In contrast, PEPCK activities were markedly lowered in those groups as summarize in Table 4.

**Table 4:** Serum and hepatic glucose-regulating enzyme activities in normal and db/db mice treated with *FYGL* for 8 weeks.

	Normal	Control	75 mg/kg *FGYL*	250 mg/kg *FYGL*	450 mg/kg *FYGL*	200 mg/kg Metformin
**Serum**						
GK (U/L)	27.23±2.3**	17.32±1.06	18.9±2.1	25.28±3.4**	25.82±4.0**	20.1±2.4*
PEPCK (IU/L)	9.34±0.18**	11.58±0.54	10.30±0.27**	10.14±0.49**	9.96±0.27**	10.02±0.18*
**Liver**						
GK (U/g protein)	4.97±0.16**	2.82±0.22	3.04±0.38	3.54±0.35*	4.64±0.28**	4.24±0.37**
PEPCK (IU/mg protein)	120.56±25.6**	295.21±35.5	289.28±42.7	204.63±29.8*	145.26±35.5**	183.58±25.1*

Results are expressed as mean ± S.E.M., n = 10.

* p<0.05 as compared to diabetic control and **p<0.01 as compared to diabetic control.

### Effects of *FYGL* on Expression of Glucose Transporter Protein

**Figure 4:**
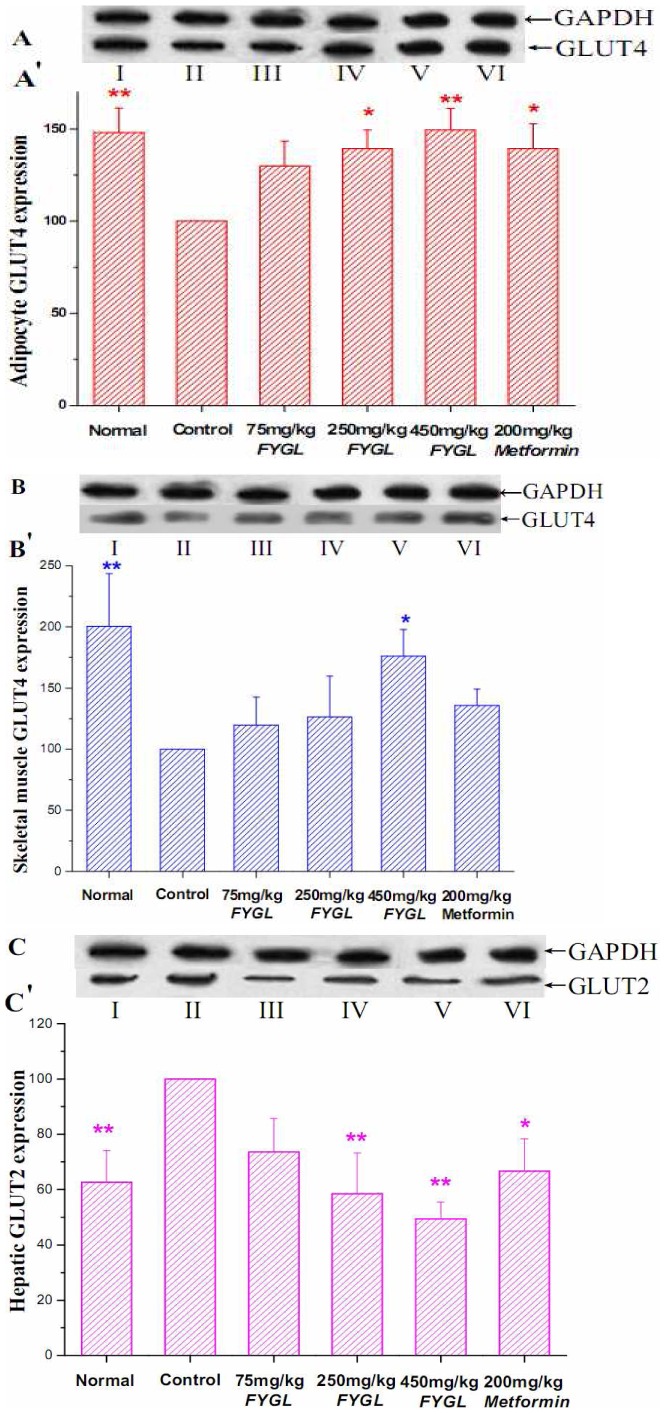
Glucose transporter protein 2 and 4 expression. Glucose transporter protein expression in normal and db/db mice after treatment for 8 weeks. (A) adipocyte GLUT4 expression; (B) skeletal GLUT4 expression; (C) hepatic GLUT2 expression. I to VI represent normal, diabetic control, diabetic treated with 75 mg/kg *FYGL*, diabetic treated with 250 mg/kg *FYGL* diabetic treated with 450 mg/kg *FYGL* and diabetic treated with 200 mg/kg metformin. Results are expressed as mean ± S.E.M., n = 10, *p<0.05 as compared to diabetic control and **p<0.01 as compared to diabetic control.

The change of hepatic GLUT2, adipose and skeletal GLUT4 protein expressions were examined by western blotting analysis. As shown in Figure 4A and 4B, the expressions of adipose and skeletal GLUT4 in diabetic control mice were decreased by 48.1% (p<0.01) and 100.5% (p<0.01), respectively, while the expression of hepatic GLUT2 (Figure 4C) was increased by 37.4%, compared to that of normal mice. High-dosage *FYGL* treatment significantly lowered the hepatic GLUT2 protein level by 51.6% compared with that of the control group. In contrast, the expressions of GLUT4 in adipose and skeletal tissues were markedly (p<0.05) increased by 49.5% and 76.2%, respectively, after high-dosage *FYGL* treatment for 8 weeks, compared with that of the control group.

### Effects of *FYGL* on Pancreas Histology

**Figure 5:**
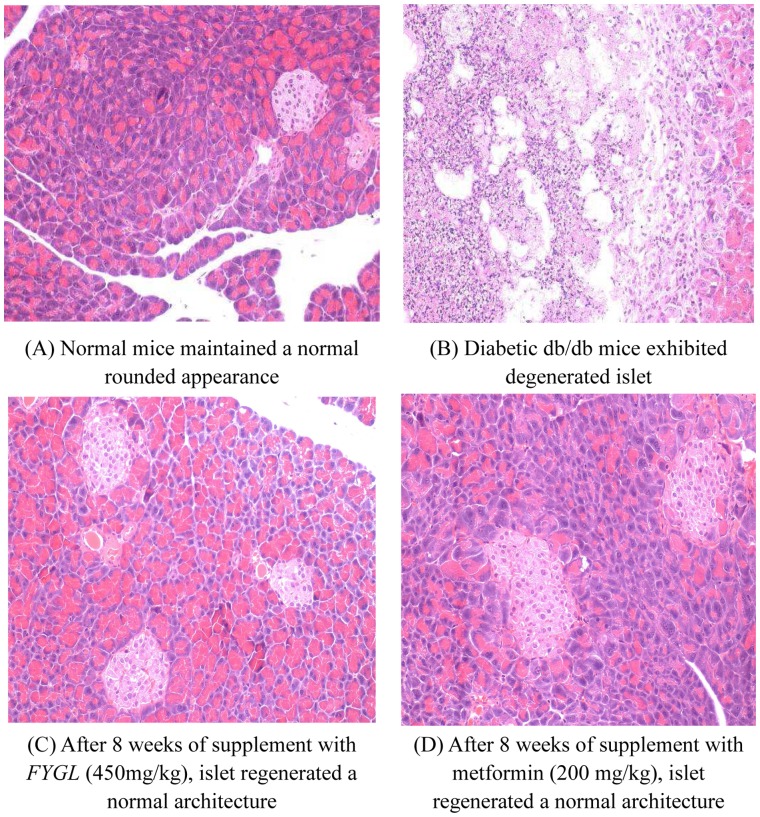
Histological observation of pancreas. Histological observation of pancreas by H&E staining. (A) Normal mice maintained a normal rounded appearance; (B) Diabetic db/db mice exhibited degenerated islet; (C) After 8 weeks of supplement with *FYGL*, islet regenerated a normal architecture; (D) After 8 weeks of supplement with metformin, islet regenerated a normal architecture.

As shown in Figure 5, after 8 weeks trial, the pancreas of normal mice maintained normal morphologies (Figure 5A). In contrast, the pancreas of diabetic control mice exhibited boundary definition loss and degeneration (Figure 5B). The pancreas of high-dosage *FYGL* and metformin treated group had also normal morphologies, suggesting the β-cell proliferation or regeneration by *FYGL* and metformin therapy (Figure 5C and 5D). The results also showed that the number of cells was increased in high-dosage *FYGL* treated group, indicating that *FYGL* may have the potential for the remediation of pancreatic islets.

## Discussion

### *FYGL* Beneficial on the Glucose Homeostasis and Pancreatic β-cells

Our previous investigation showed the potent antihyperglycemic activity of a novel proteoglycan, named *FYGL*, from *G. Lucidum* fruiting bodies. In the present study, we attempted to figure out whether *FYGL* had more function such as hypolipidemic and antioxidant in DM. This study showed that the supplementation of *FYGL* for eight weeks improved the glucose homeostasis in db/db mice, in agreement with our previous results [13], [14]. DM patients exhibit a marked reduction in insulin-mediated glucose disposal. Poor control of postprandial glucose, as a marker of risk for macro-vascular complications, is associated with elevated rates of all-cause mortality, making postprandial glucose being more important than fasting glucose. The present study observed that the supplementation of *FYGL* for eight weeks can significantly improve impaired glucose tolerance in db/db mice. Furthermore, the *FYGL* supplements significantly lowered the HbAlc level, a useful parameter in the monitoring of long-term blood glucose. Thus, *FYGL* effect on glucose homeostasis solidly confirmed its antihyperglycemic activity.

In db/db mice, progressive impairment of insulin response to chronic hyperglycemic conditions is known to be age-dependent. The variation caused by the insulin resistance in the early stage is islet β-cell hyperplasia resulting in marked hyperinsulinemia. However, when the db/db mice reach at 12 to 24 weeks old, islet develops β-cell necrosis, hyperinsulinemia is diminished, and the mice manifest symptoms of insulin deficiency [29]. As we observed in present work, at the end of experiments, i.e., when the mice were 16 weeks old, the insulin level of db/db mice was much lower than the normal mice. *FYGL* treatment also increased the C-peptide levels which has a longer half-life than insulin and thus can represent insulin secretion more accurately than the insulin levels do. Furthermore, we also observed that the pancreas of db/db mice exhibited boundary definition loss and degeneration, while 8 weeks *FYGL* trials remarkably alleviate those symptoms indicating that *FYGL* can preserve the pancreas architecture. Taken together, the present data suggested that the serum insulin level in the db/db mice may be gradually declined after reaching the peak point due to the reduction of β-cell mass, whereas *FYGL* is capable to slow the age-dependent insulin secretion decline, i.e., *FYGL* could partly protect the pancreas from dysfunction which is associated with impaired insulin secretion and biosynthesis. Similar effects of natural products on pancreas of db/db mice were also reported in Jung’s research [30], in which they investigated the hypoglycemic effect of caffeic acid in db/db mice and demonstrated that the plasma insulin levels in caffeic acid-treated group were significantly enhanced due to an anti-degenerative effect of caffeic acid on the islets.

Insulin plays a critical role in clearing the postprandial glucose load and in maintaining hepatic glucose homeostasis, by activating glycogen synthesis and glycolysis whereas inhibiting gluconeogenesis. As we observed, the hepatic glycogen content of diabetic mice were obviously lower than those of the normal mice. It is interesting that all those values were significantly enhanced after *FYGL-*treated for eight weeks and they were almost similar to those of metformin supplemented mice. Metformin has been reported to enhance insulin action, thereby improving glucose tolerance in animals and humans with DM [31]. The concomitant increase in insulin and the improvement of glucose tolerance suggested that *FYGL* may enhance insulin secretion in the late stages of the DM. Taken together, these data suggested that *FYGL* may have a potent of pancreas protection thus leading to a drastic increase of DM alleviation.

### *FYGL* Beneficial on Dyslipidemia, Obesity, and Oxidative Stress

It was reported that diabetes was associated with profound alterations in lipid and lipoprotein profiles. In addition to its hypoglycemic effect, *FYGL* had strong hypotriglyceridemic and hypocholesterolemic effects on db/db mice with a significant reduction in serum LDL-C levels and an increase in HDL-C levels. Furthermore, the atherogenic indexes (AI) of *FYGL*-treated mice were markedly decreased, leading to a reduction in LDL/HDL ratio. The increase in LDL/HDL ratio is one of the most important criteria for the anti-atherogenic agents. The relative high levels of HDL-C are associated with low incidences of vascular diseases and complications [32]. Thus, our results suggested that *FYGL* could be helpful for the prevention of diabetic complications through improving dyslipidemia.

Moreover, there is no significant difference in the final body weight and body weight gain trend between the diabetic control mice and the *FYGL*-treated diabetic mice, indicating that *FYGL* would not result in overweight gain perhaps due to its decreasing effect on lipogenic enzyme activity such as fatty acid. Meanwhile, the fact that the GLUT4 protein expression in both adipose and skeletal muscle was siginicantly enhanced by *FYGL* treatment may be also related to the anti-obesity effect of *FYGL* since increase of adipose and skeletal muscle glucose disposal can attribute to the alleviation of fat accumulation.

Obesity is the strongest predictor in the development of DM. The relationship between obesity and DM has drawn much attention for a long time and it has been recently proposed that oxidative stress may be a primary factor in the etiology of obesity-induced DM [33]. Oxidative stress caused from excess reactive oxygen species (ROS) which could react with polyunsaturated fatty acids, can lead to the lipid peroxidation. Increased lipid peroxidation impairs membrane function by decreasing membrane ﬂuidity and changing the activity of membrane-bound enzymes and receptors [34], [35]. Normally, antioxidant defense system can produce enough scavengers such as SOD, CAT, and GSH-Px to protect against injury by ROS. However, diabetics usually exhibit high oxidative stress due to persistent and chronic hyperglycemia, which thereby reduces the total antioxidant activities and thus promotes the generation of ROS [36]. As an end product of lipid peroxidation, malondialdehyde (MDA) content reﬂects the degree of the whole lipid oxidation in body; and the prolonged exposure of protein to ROS could lead to the conversion of protein to carbonyl derivatives spontaneously, thus, the protein carbonyls (PC) formed are considered sensitive indices of oxidative injury to proteins [37]. Additionally, DNA is susceptible to oxidation, resulting in the formation of 8-hydroxyguanosine (8-OHdG) which is a relatively stable oxidation product and can accurately represent DNA damage from oxidative stress. In the present study, the drastic increases of MDA, 8-OHdG and PC level were observed in liver of diabetic db/db mice, indicating the oxidation stress occurred in diabetic mice. However, administration of *FYGL* significantly alleviated these symptoms. The levels of MDA, 8-OHdG and PC in *FYGL* treated db/db mice were definitely lower than that in the control mice and administration of *FYGL* also lead to a significant increase in the antioxidant enzyme activities, including SOD, CAT and GSH-Px, in both serum and liver. Hence, it was also possible that the antidiabetic ability of *FYGL* may be related to its antioxidant activity.

### *FYGL* Effect on Insulin Signaling Transduction Pathway

The insulin action is regulated and initiated by its receptor (IR). When insulin binds to its receptor, changes in the intracellular conformation of the receptor result in the O-phosphorylation of specific tyrosine residues. This serves as the first step in insulin signaling, and it is followed by a cascade of intracellular events which mediate the physiological effects of insulin, such as glucose uptake [38]. There is compelling evidence that PTP1B, is primarily responsible for the dephosphorylation of the insulin receptor, consequently, leading to the block of insulin signaling transduction pathway. Thus, a PTP1B inhibitor would be expected to increase the half-life of the phosphorylated insulin receptor and enhance the effects of insulin [39]. Researches *in vivo* indicated that PTP1B knock-out mice were found to be very healthy and exhibited increasing insulin sensitivity [40]. Therefore, searching for an effective inhibitor of PTP1B has been considered worthwhile for developing drugs for the treatment of DM. *FYGL* was an efficient PTP1B inhibitor with IC_50_ of 5.12±0.05 ug/ml as we demonstrated before [12]–[14]. A decrease in the tyrosine phosphorylation levels of the IR β-subunit in the liver and skeletal muscle of diabetic animals was also observed in our previous work, implying the PTP1B role in diabetes. Interestingly, previous investigation in our lab indicated that four weeks *FYGL*-treated animals, including STZ-induced diabetic rats and C57BL/6 db/db mice, exhibited an decrease PTP1B expression and activity in liver and skeletal muscle. Furthermore, the increase in the tyrosine phosphorylation level of the IR of four weeks *FYGL*-treated animals was also observed previously, indicating the improvement of insulin sensitivity by *FYGL* treatment in the early stage of DM. So far, no PTP1B inhibitor or such as *FYGL* has been found to be effective in multi-tissues *in vivo*.

### *FYGL* Effect on Glucose-regulating Enzyme Activity

Many phytochemicals were reported to have multiple functions *in vivo*. Thus, the effects of *FYGL* on glucose-regulating enzyme activity, which were known to be altered in diabetics, were also investigated systematically in present. GK, as one of the key glucose-regulating enzymes in the catabolism of glucose, can phosphorylate glucose and convert it into glucose-6-phophate. GK is a potential target for pharmacological treatment of DM, as evidenced by the fact that GK-knock out mice exhibited mild hyperglycemia, while the over expression of GK lead to a relative lower blood glucose concentration in the diabetic models [41].The antihyperglycemic action of *FYGL* was also probably associated with the GK activity as we observed that the hepatic GK activity of diabetic mice was significantly lower than that of the normal mice, whereas supplement of *FYGL* increased the GK activity in a dose-dependent manner. The increase of hepatic GK activity can increase the utilization of the blood glucose for energy production or glycogen storage [42]. As we observed that the db/db mice present a significantly increase in hepatic glycogen contents when supplemented with *FYGL*. In contrast to GK, the PEPCK is a key rate-limiting enzyme which is important in the regulation of gluconeogenesis. Chronic hyperglycemic conditions would lead to an increase in PEPCK expression, while inhibition of the PEPCK expression can reduce the blood glucose levels and improved glucose tolerance together with a decreased lipid levels in the diabetic mice. On the other hand, the over expression of PEPCK can result in hyperglycemia, hyperinsulinemia, and impaired glucose tolerance leading to a vicious cycle in the diabetic patients, in which both the gluconeogenesis and PEPCK are found to be up-regulated [43]. In our study, the hepatic PEPCK activity in diabetic mice was significantly higher than that in normal mice which maybe responsible for or associated with the elevated lipid levels, poor glucose tolerance and low hepatic glycogen content, while *FYGL* consumption caused a marked suppression of the hepatic PEPCK activity. Consequently, *FYGL* seemed to be able to suppress the hepatic glucose output by enhancing hepatic glucose utilization and inhibiting glucose over production in diabetic mice.

### *FYGL* Effect on Glucose Transporter Protein Expression

The most important effect of insulin is the stimulation of glucose transport in cells and the rate-limiting step in the uptake and metabolism of glucose by insulin target cells is glucose transport, which is mediated by specific glucose transporters of the plasma membrane. Thus, along with the measurement of GK and PEPCK activities, the hepatic GLUT2, skeletal muscle and adipocytes GLUT4 expression were also taken account in the present study. A major metabolic defect associated with DM is the failure of peripheral tissues to properly utilize glucose, thereby resulting in chronic hyperglycemia. Generally, Hepatic Glut2 expression is higher in obese and diabetic animals, such as db/db mice [44]. As we observed in present work, the hepatic GLUT2 expression was marked higher in the diabetic mice, compared to the normal group. Supplement of *FYGL* for eight weeks, the GLUT2 expression was significantly decreased, which is known to be related with a decrease in hepatic glucose output [45]. GLUT4, the major insulin-dependent transporter, presents predominantly in skeletal muscle and adipose tissue and plays an important regulation role in whole-body glucose homeostasis [46]. Skeletal GLUT4 over expression is known to alleviate insulin resistance and pancreatic defects in db/db mice, resulting in a markedly improved glycemic control. The present study showed that *FYGL* significantly enhanced the GLUT4 protein expression in both adipose and skeletal muscle tissue, compared with the diabetic control mice.

In conclusion, the data obtained from this present study suggest that *FYGL* was an effective antidiabetic agent via its ability to enhance insulin sensitive and to decrease hepatic glucose output along with the increased level of adipocyte and skeletal muscle glucose disposal in the DM animals. Furthermore, it seems likely that *FYGL* is beneficial against oxidative stress, thereby being helpful in preventing or delaying the development of diabetes and its complications.
